# Supplementary material for: Developmental function and state transitions of a gene expression oscillator in Caenorhabditis elegans
Source: Mol Syst Biol. 2020 Jul 20;16(7):e9498. doi: 10.15252/msb.20209498 (PMC7370751; doi:10.15252/msb.20209498)
Supplement: Supplementary file 1 — Appendix [file MSB-16-e9498-s001.pdf]

# Appendix

## Developmental function and state transitions of a gene expression oscillator in *C. elegans*

**Authors:** Milou W.M. Meeuse, Yannick P. Hauser, Lucas J. Morales Moya, Gert-Jan Hendriks, Jan Eglinger, Guy Bogaarts, Charisios Tsiairis, Helge Großhans*

*Correspondence to: helge.grosshans@fmi.ch.

**This PDF file includes:**

Figs. S1 to S8
Tables S1 to S2
Supplementary text
References

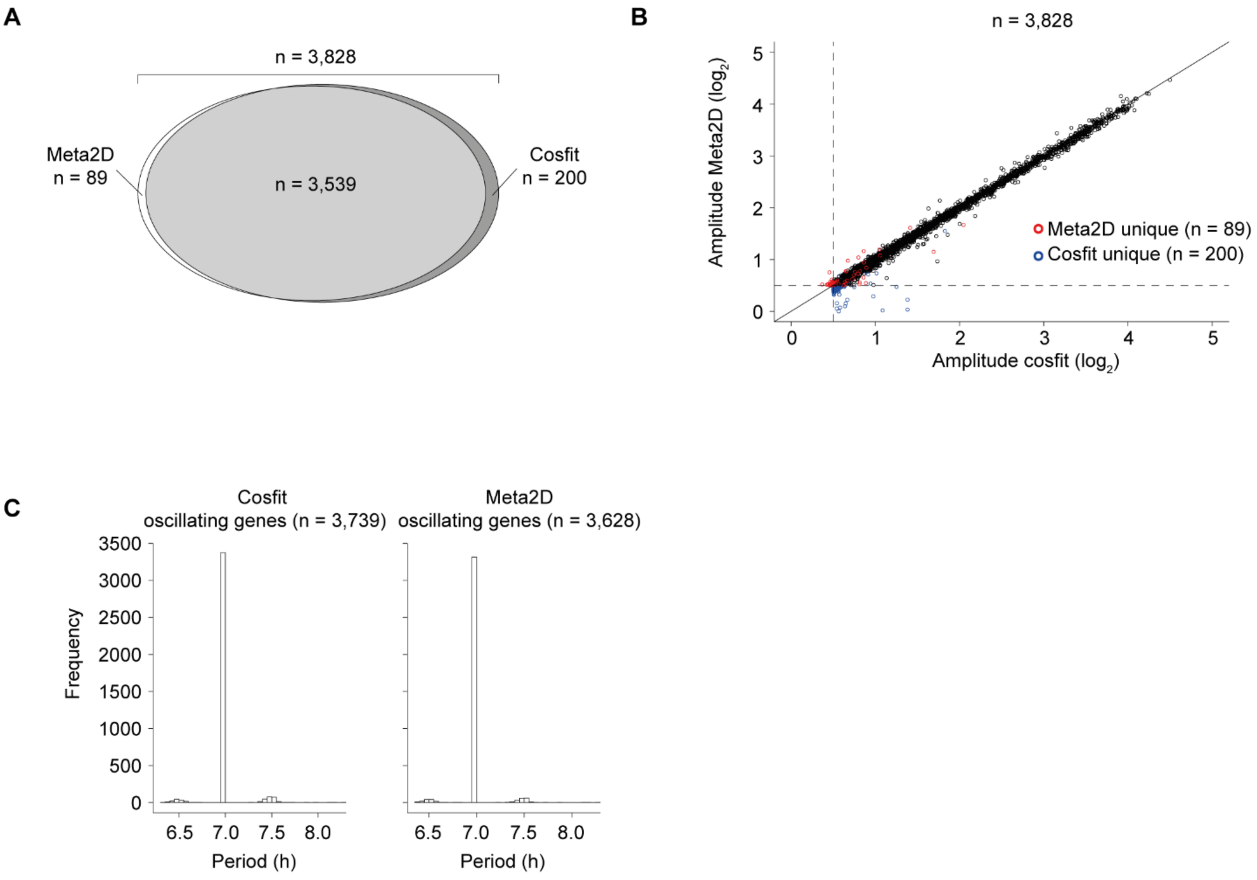


**Fig. S1: Comparison of Cosine fitting and MetaCycle in identification of oscillating genes**
The function Meta2D of the R package MetaCycle (Wu *et al*, 2019, 2016) was used to identify oscillating genes (with FDR<0.05 and $\log_2$(amplitude) $\geq$ 0.5) and compared with genes identified by Cosine fitting ($\log_2$(amplitude) $\geq$0.5 and p$\leq$0.01, Fig. 1 and Fig. EV1).
**(A)** Venndiagram showing the genes identified as oscillating in both Meta2D and Cosine fitting (Cosfit) methods (n=3,539) and oscillating genes uniquely identified in Meta2D (n=89) or Cosine fitting (n=200) respectively.
**(B)** Scatterplot of amplitude determined by Meta2D over amplitude determined by Cosine fitting. Oscillating genes uniquely identified by Meta2D and Cosine fitting are indicated in red and blue respectively.
**(C)** Histogram of oscillation period determined by Meta2D method for oscillating genes identified by Cosine fitting (left) and by Meta2D (right) methods.

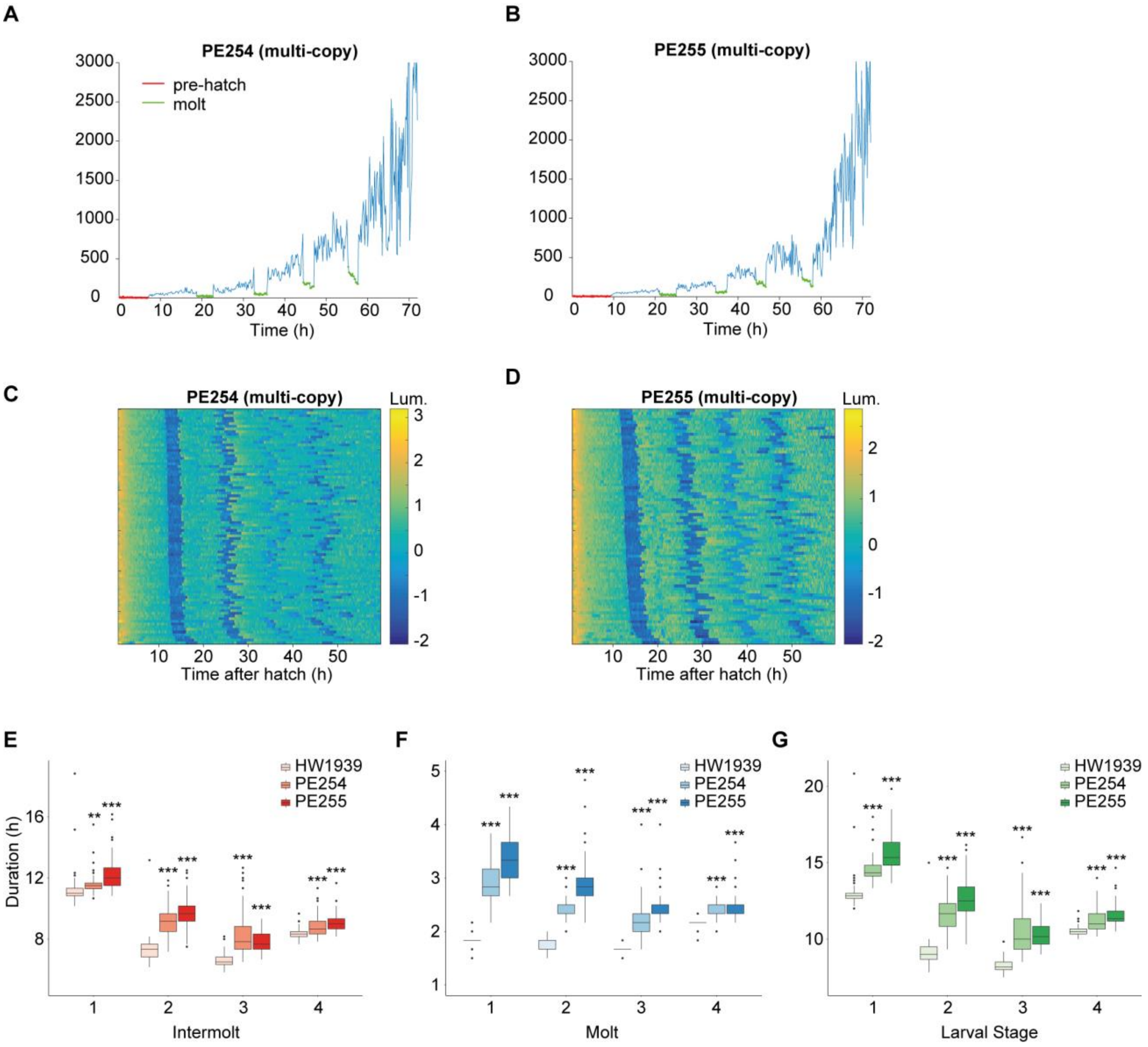


H

| | stage 1 | stage 2 | stage 3 | stage 4 |
|---|---|---|---|---|
| Intermolt PE254 | 1.04 (p=3.28E-03) | 1.26 (p=4.51E-31) | 1.26 (p=3.07E-19) | 1.05 (p=1.94E-08) |
| Intermolt PE255 | 1.10 (p=1.98E-10) | 1.32 (p=1.39E-35) | 1.19 (p=3.72E-30) | 1.08 (p=2.10E-16) |
| Molt PE254 | 1.60 (p=4.92E-51) | 1.40 (p=9.60E-54) | 1.33 (p=2.11E-29) | 1.11 (p=8.68E-23) |
| Molt PE255 | 1.84 (p=2.49E-50) | 1.65 (p=3.53E-37) | 1.46 (p=2.33E-38) | 1.15 (p=9.74E-21) |
| Larval stage PE254 | 1.11 (p=5.20E-20) | 1.28 (p=2.35E-40) | 1.27 (p=1.99E-22) | 1.07 (p=4.32E-14) |
| Larval stage PE255 | 1.20 (p=6.93E-31) | 1.38 (p=2.97E-42) | 1.25 (p=3.97E-40) | 1.10 (p=8.24E-22) |

**Fig. S2: A strain with single-copy integrated luciferase transgene develops rapidly and synchronously**

**(A-B)** Representative raw luminescence traces of individual animals grown at 20°C. As the egg-shell is impenetrable to luciferin, a sudden increase in luminescence at the beginning of the time course indicates hatch (pre-hatch in red). Abrupt drops and subsequent rises in luminescence specify molts (in green). The previously published strains (A, PE254; B, PE255) (Olmedo *et al*, 2015) express luciferase from randomly integrated multi-copy transgene arrays that carry a semi-dominant version of the cuticular collagen *rol-6* as a marker (Lagido *et al*, 2008). To exclude that

this genetic make-up could interfere with our quantification, we integrated a luciferase transgene, driven by the strong, ubiquitous and constitutive *eft-3* promoter, into the genome through Mos1-mediated single copy integration (MosSCI) (Fig. EV2, HW1939).
**(C-D)** Heatmap per strain showing trend-corrected luminescence (Lum.) trace for one animal per horizontal line (C, Multi-copy integrated PE254 (n=88). D, Multi-copy integrated PE255 (n=79)). Hatch is set to t = 0 h and traces are sorted by time of entry into first molt. Blue indicates low luminescence and corresponds to the molts.
**(E-G)** Quantification of the duration of each intermolt (E), molt (F), larval stage G) for indicated strains in hours. The newly generated strain developed more rapidly and with less variability with regard to the duration of individual stages. Although the general trend in larval stage durations was shared between the different strains, i.e. L1>L4>L2&L3 (G), animals carrying the *rol-6*-marked multi-copy luciferase arrays also exhibited an extended M1 molt (F) as reported previously (Olmedo *et al*, 2015). This effect disappeared when using the single-copy transgene strain. Hence, the duration of molt M1 became comparable to that of M2 and M3 and lengthening of L1 is explained by lengthening of intermolt 1. Significant differences between single-copy integrated (n=86) and multi-copy integrated strains (PE254 (n=88) and PE255 (n=79)) is indicated (**, P<0.01, *** P<<0.001, Welch two sample, two-sided t-test). Boxplots extend from first to third quartile with a line at the median, outliers are indicated with a cross, whiskers show 1.5*IQR.
**(H)** Table showing fold changes of mean durations of indicated stages for PE254 and PE255 compared to HW1939 for data shown in E-G. P-values are indicated in brackets (Welch two-sample, two-sided t-test).

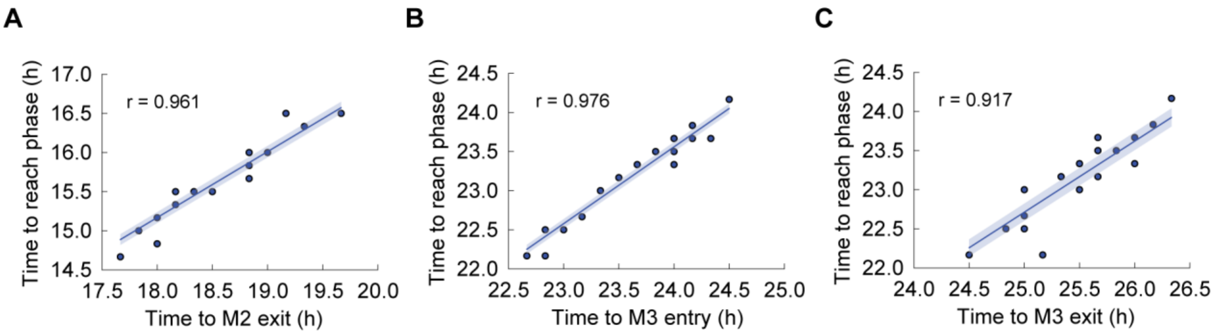


**Fig. S3: Durations to reach molts and an arbitrarily chosen phase correlate highly**
Additional scatterplots comparing the absolute times needed for M2 exit (A), M3 entry (B) and M3 exit (C) with the time required to reach a arbitrarily chosen GFP oscillation phase for *qua-1p::gfp::pest::h2b::unc-54*$_{3'UTR}$ expressing animals (HW2523) investigated in the single worm imaging (Fig 3F). The arbitrary phase was chosen at the end of the respective larval stages. Pearson correlation coefficients (r) all exceeded 0.9.

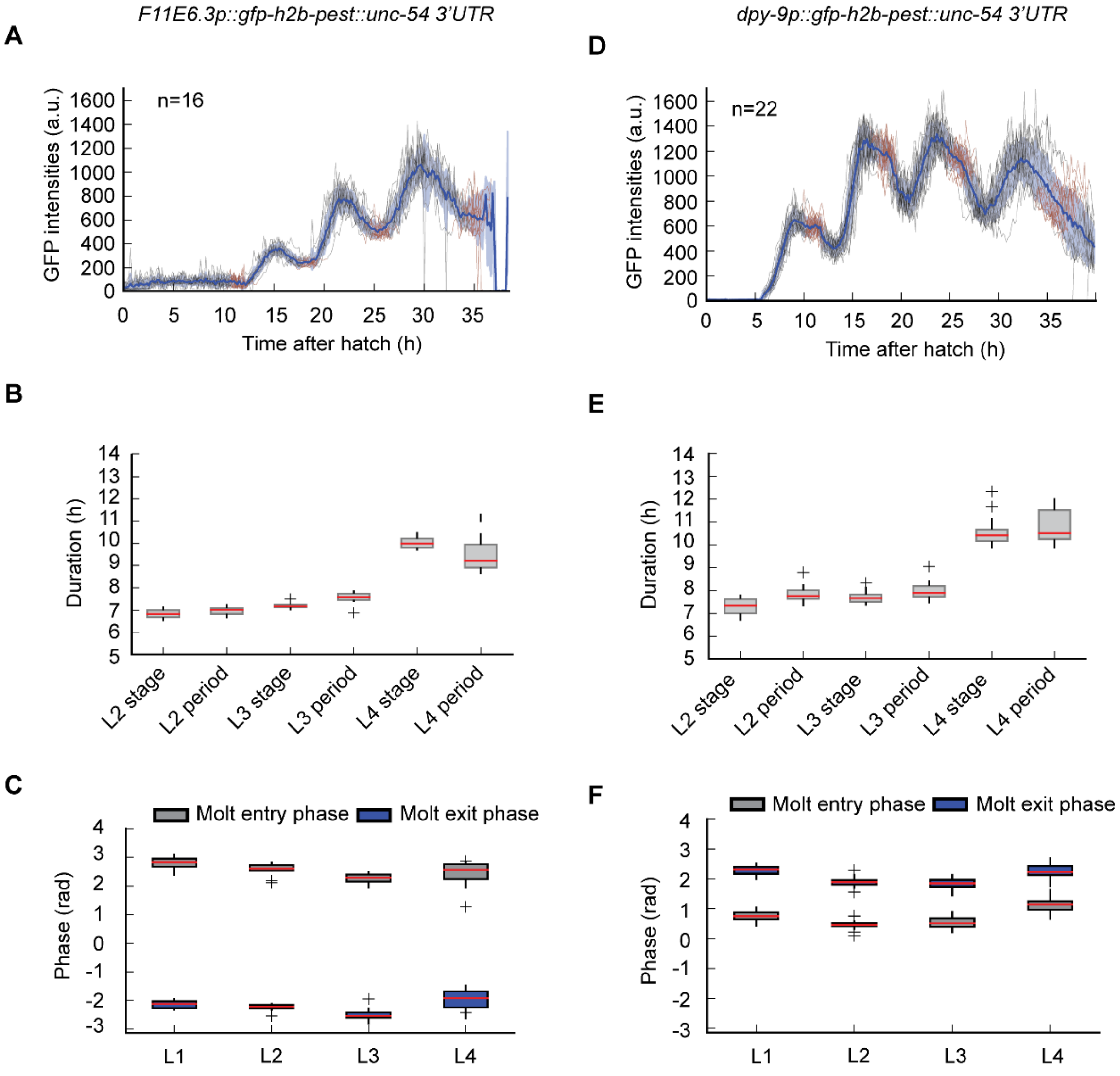


**Fig. S4. Single worm imaging with two additional reporter strains confirms phase-locking of oscillations to molts independently of peak phases**

**(A, D)** GFP quantification of single worm kymographs for the *F11E6.3* (HW1370, n=16) and the *dpy-9* (HW2526, n=22) transcriptional reporters respectively. All traces of individual worms were aligned to the time of hatching, which was set to t = 0 h. The traces are colored in black during intermolts and in red during molts. The blue shading indicates the standard deviation of the population at each time point with the blue line representing the mean across the worm population. Only three peaks are visible for the *F11E6.3* reporter, because the assay terminated before the final rise in expression seen with RNA sequencing.

**(B, E)** Comparison of larval stage duration and period times of oscillations in hours for L2-L4 larval stages for *F11E6.3* and *dpy-9* transcriptional reporters respectively.

**(C, F)** Boxplot of expression phases at molt entry (start of lethargus) and molt exit (end of lethargus) separated by larval stages; n = 16 for *F11E6.3* (D) and n = 22 for *dpy-9* (I) transcriptional reporters.

Boxplots extend from first to third quartile with a line at the median, outliers are indicated with a cross, whiskers show 1.5*IQR.

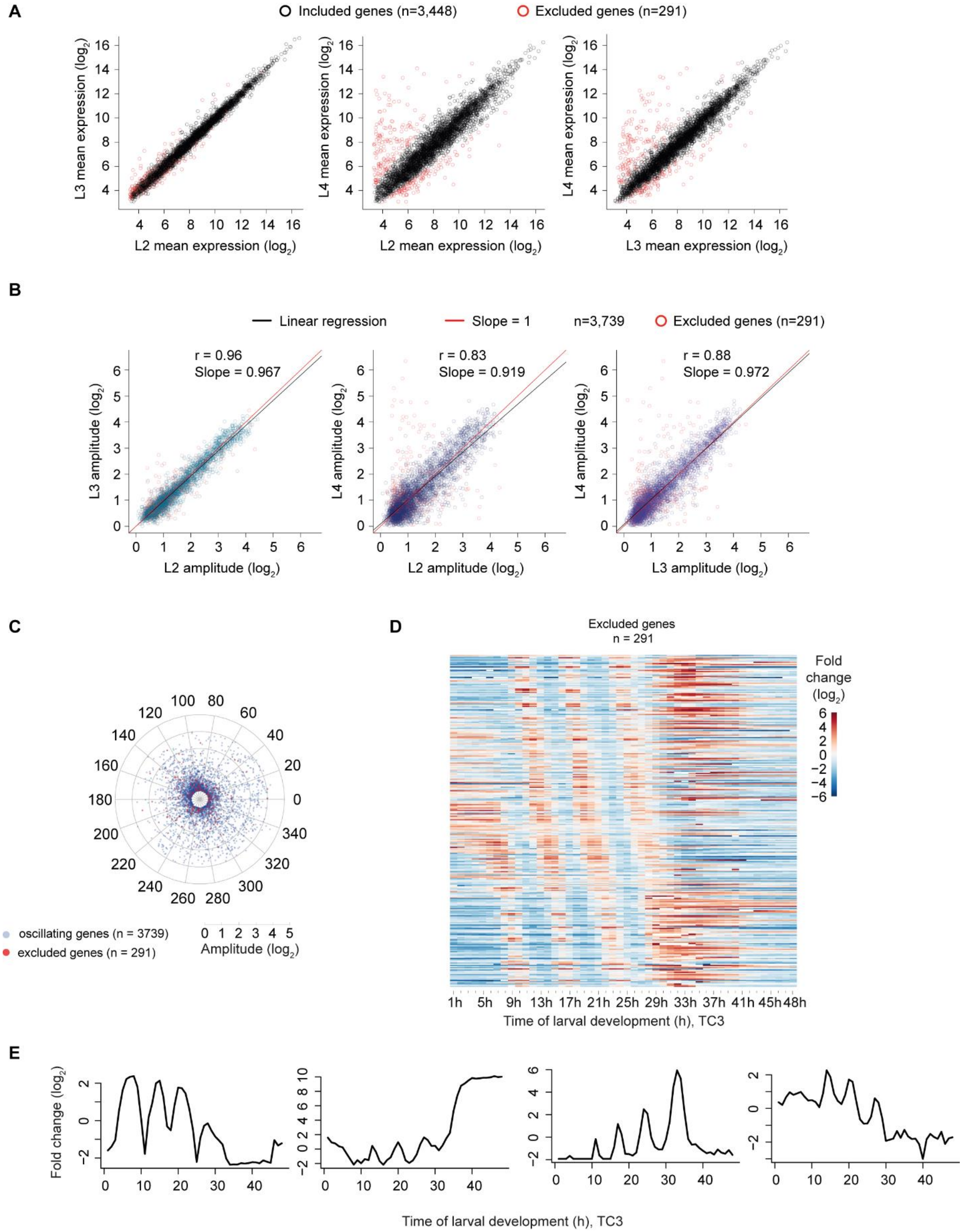
A
Included genes (n=3,448)
Excluded genes (n=291)
L3 mean expression (log2)
L2 mean expression (log2)
L4 mean expression (log2)
L2 mean expression (log2)
L4 mean expression (log2)
L3 mean expression (log2)
B
Linear regression
Slope = 1
n=3,739
Excluded genes (n=291)
r = 0.96
Slope = 0.967
r = 0.83
Slope = 0.919
r = 0.88
Slope = 0.972
L3 amplitude (log2)
L2 amplitude (log2)
L4 amplitude (log2)
L2 amplitude (log2)
L4 amplitude (log2)
L3 amplitude (log2)
C
oscillating genes (n = 3739)
excluded genes (n = 291)
Amplitude (log2)
D
Excluded genes
n = 291
Fold change (log2)
Time of larval development (h), TC3
E
Fold change (log2)
Time of larval development (h), TC3

**Fig. S5: Exclusion of genes based on deviating behavior in L4 stage**

**(A)** Scatter plot showing mean expression over time in L2, L3 or L4 for each oscillating gene. Genes indicated in red were excluded based on the L2-L4 scatter plot (Methods).

**(B)** Scatter plot showing the amplitude in L2, L3 or L4 for each oscillating gene. Genes indicated in red correspond to red genes in A and were excluded from amplitude and period analysis in Fig. 4.

**(C)** Polar Scatterplot visualizing the amplitude and peak phase of all oscillating genes (n = 3,739) in blue and the excluded oscillating genes (n = 291) in red. The excluded genes do not show a particular peak phase or amplitude preference.

**(D)** Gene expression heatmap of $log_2$ transformed mean normalized data of the excluded oscillating genes in the fused time course TC3 (n = 291).

**(E)** Example gene expression of four excluded oscillatory genes that were excluded based on the L2-L4 scatter plot in (A).

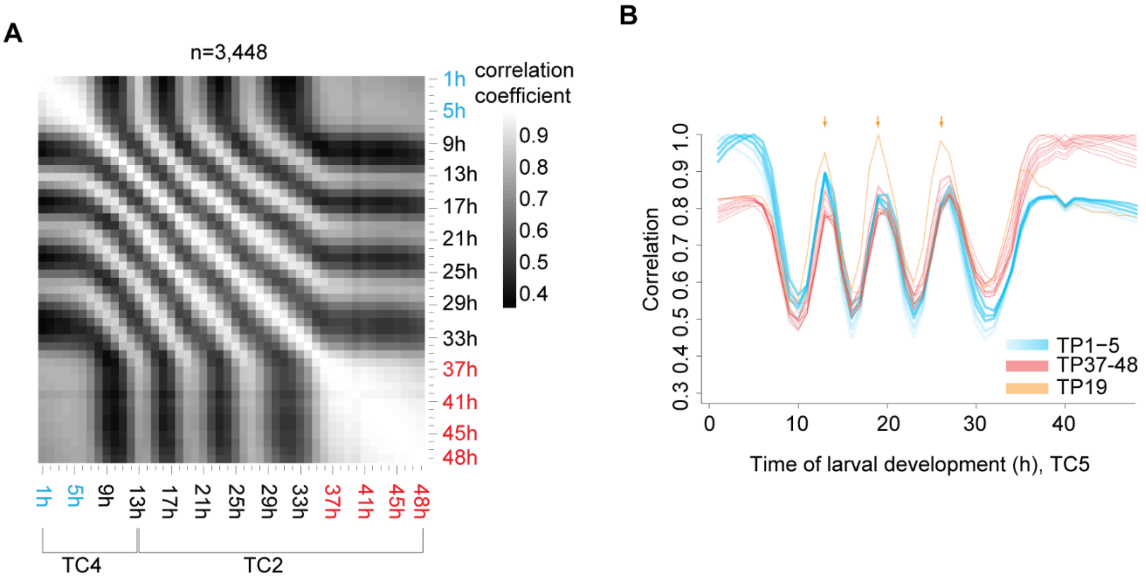


**Fig. S6: Arrested phase of the oscillator is reproduced in a second RNA sequencing experiment**

**(A)** Pairwise correlation plot of $log_2$-transformed oscillatory gene expression patterns without L4 deviating genes (n=3,448) from the replicate time course TC4 (TP1 – 13) fused with TP14 – 48 of the long RNA seq time course TC2 (TP14 – 48).

**(B)** Correlations of expression patterns for the indicated time points to all other time points of the fused time course from A. TP1 – 5 (blue) as well as the adult time points (red) correlate highly with TP13, TP19 and TP26 (arrows). Hence, oscillations are arrested in the same phase at the beginning and the end of the time course.

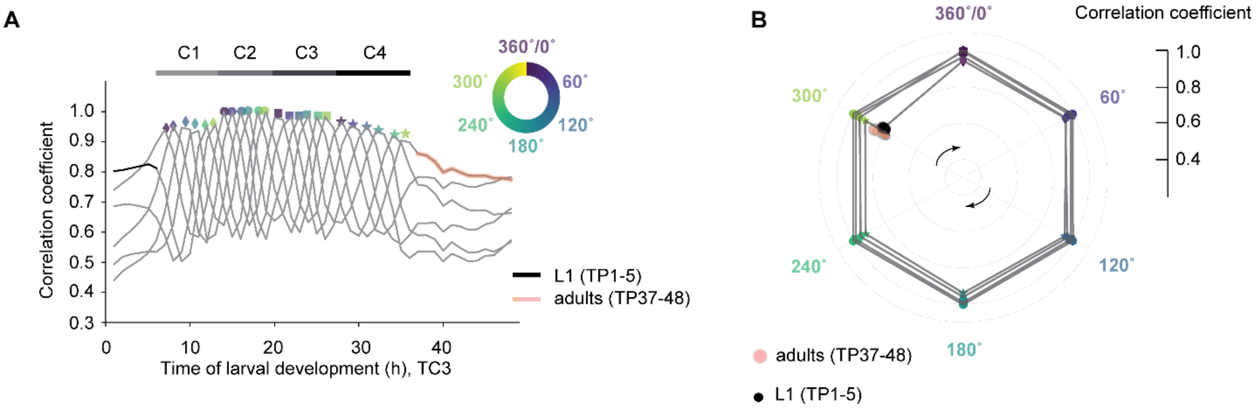


**Fig. S7: Oscillations are invariant over time**

The correlation analysis revealed a stably arrested oscillator state in early L1 and young adults. To explore how the oscillating state changes over time, we investigated the similarity among the four oscillation cycles, C1 through C4. Specifically, we compared oscillator states at each time point sampled during C2 to the three other cycles. By choosing C2 as a starting point, we could examine correlations to both earlier and later cycles. Because the last time point (360°) of one cycle is the first time point (0°) of the following cycle, we truncated each cycle at 300° for this analysis, to avoid an artificially inflated correlation.

Using spline interpolation and local maxima detection to determine correlation peaks (Methods) for each of the six time points TP14/0° through TP19/300° of C2 to the other cycles, we observed high and largely invariant values across each of the other three cycles. In other words, except for the extended period during L4, little variability occurs in oscillations across the four cycles.

**(A)** Correlation of cycle 2 time points (TP14–19; corresponding to 0° to 300°; marked by indicated colors) to all other time points of the fused larval time course (TC3). For the correlation analysis we used the $\log_2$-transformed oscillating gene expression data without the L4 deviating genes. Diamonds (cycle 1), circles (cycle 2), squares (cycle 3) and stars (cycle 4) indicate correlation peak values and peak times determined by spline interpolation.

**(B)** Polar plot displaying correlation of cycle 2 gene expression patterns with those of the corresponding points in the other cycles. Color scheme and symbols as in B. Adult time points (red circles) and start (0° in cycle 1; orange) are placed according to correlations in A and B, respectively.

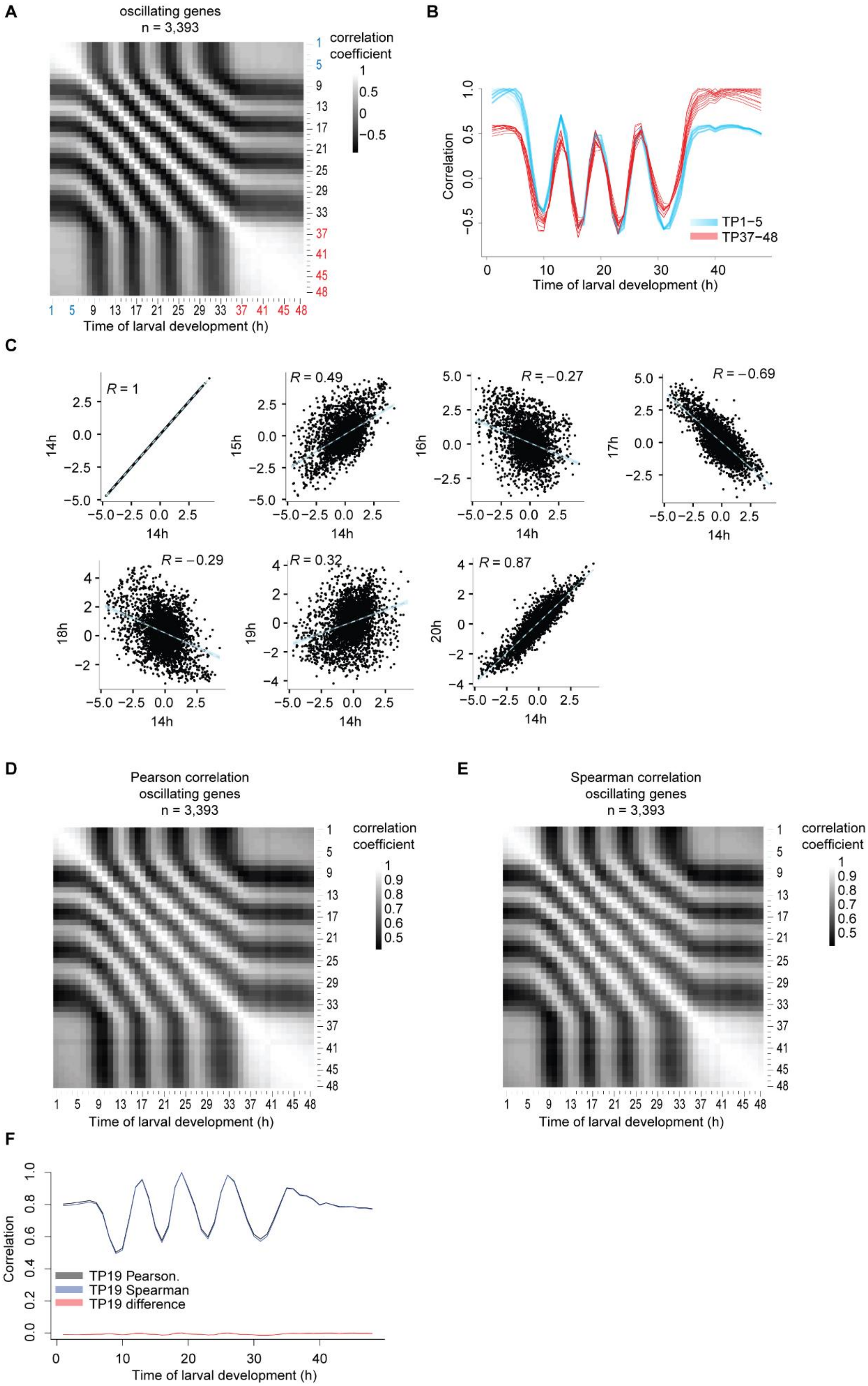
A
oscillating genes
n = 3,393
correlation
coefficient
1
0.5
0
−0.5
Time of larval development (h)
B
Correlation
TP1−5
TP37−48
Time of larval development (h)
C
R = 1
R = 0.49
R = −0.27
R = −0.69
R = −0.29
R = 0.32
R = 0.87
14h
15h
16h
17h
18h
19h
20h
D
Pearson correlation
oscillating genes
n = 3,393
correlation
coefficient
Time of larval development (h)
E
Spearman correlation
oscillating genes
n = 3,393
correlation
coefficient
Time of larval development (h)
F
Correlation
TP19 Pearson.
TP19 Spearman
TP19 difference
Time of larval development (h)

**Fig. S8: Correlation analysis with mean normalized data yields qualitatively similar results to an analysis without mean normalization**

**(A)** Pairwise correlation plot of $log_2$-transformed, mean normalized oscillating gene expression from the fused developmental time course (Fig 5B, n = 3,393).
**(B)** Correlation line plots reveal repetitive similarity of TP1 – 5 to TP13, TP19 and TP26. Due to mean normalization, the correlation lines oscillates around 0.
**(C)** Scatter plot comparing $log_2$-transformed, mean-normalized oscillating gene expression data of individual time points. The Spearman correlation coefficient is indicated on the left corner and can range from anti-correlation (-1) to full correlation (+1).
**(D), (E)** Pairwise correlation plot of $log_2$-transformed oscillating gene expression from the fused developmental time course (Fig 5B, n = 3,393) using either Pearson (D) or Spearman (E) correlation.
**(F)** Correlation line plot comparing Pearson and Spearman correlation of TP19 with all other time points. The difference is small and indicated in red.

**Table S1. Related to Figure 3 and Figure S4 - Larval developmental duration in single worm imaging experiments**

Median durations of molts, intermolts and larval stage durations determined for single worm imaging reporter strains grown in microchambers at ~21°C ambient temperature.

| Reporter | Larval stage | Median duration (h) |
|---|---|---|
| qua-1 | I1 | 10 |
| qua-1 | I2 | 4.7 |
| qua-1 | I3 | 5 |
| qua-1 | I4 | 6.8 |
| qua-1 | M1 | 2.2 |
| qua-1 | M2 | 1.8 |
| qua-1 | M3 | 1.8 |
| qua-1 | M4 | 2.8 |
| qua-1 | L1 | 12.2 |
| qua-1 | L2 | 6.5 |
| qua-1 | L3 | 6.9 |
| qua-1 | L4 | 9.5 |
| dpy-9 | I1 | 10.2 |
| dpy-9 | I2 | 5.5 |
| dpy-9 | I3 | 5.6 |
| dpy-9 | I4 | 7.8 |
| dpy-9 | M1 | 2 |
| dpy-9 | M2 | 1.8 |
| dpy-9 | M3 | 2 |
| dpy-9 | M4 | 2.7 |
| dpy-9 | L1 | 12 |
| dpy-9 | L2 | 7.3 |
| dpy-9 | L3 | 7.7 |
| dpy-9 | L4 | 10.4 |
| F11E6.3 | I1 | 10.5 |
| F11E6.3 | I2 | 5 |
| F11E6.3 | I3 | 5.3 |
| F11E6.3 | I4 | 7.5 |
| F11E6.3 | M1 | 1.8 |
| F11E6.3 | M2 | 1.7 |
| F11E6.3 | M3 | 1.8 |
| F11E6.3 | M4 | 2.5 |
| F11E6.3 | L1 | 12.5 |
| F11E6.3 | L2 | 6.8 |
| F11E6.3 | L3 | 7.2 |
| F11E6.3 | L4 | 10 |

## Table S2. Plasmids and primers used

| Vector name | Backbone | Inserts | Primers | Primer sequence |
|---|---|---|---|---|
| pYPH0.14 | pCFJ150 | GFP::H2B::Pest | GFP-pest-H2B FW1 + Overhang | gcgtgtcaataatatcactcGCTAGCATGTCTAGACTTAGCCATGGC |
| | | | GFP-pest-H2B RV1 + Overhang | gccgatgcggagctcttatcTTACTTGCTGGAAGTGTACTTG |
| | | Unc-54 3'UTR | Unc-54 3’UTR FW1 + Overhang | AGTACACTTCCAGCAAGTAAgataagagctccgcatcg |
| | | | Unc-54 3’UTR RV1 + Overhang | Aacatatccagtcactatggaaacagttatgtttggtatattggga |
| pYPH5 | pYPH0.14 | F11E6.3 promoter | F11E6.3 FW1 + Overhang | gcgtgtcaataatatcactcaggaaaacctcaaattttgttaacact |
| | | | F11E6.3 RV + Overhang | GCTAAGTCTAGACATcatggttacctaaaaatataaagctct |
| pYPH69 | pYPH0.14 | dpy-9 promoter | dpy-9 promoter FW +OH to pYPH0.14 | gcgtgtcaataatatcactcgtacaatagaaaaaaagcagcaat |
| | | | dpy-9 promoter RV +OH to pYPH0.14 | CCATGGCTAAGTCTAGACATtctgcaataataagtattgaaaacaaga |
| pYPH70 | pYPH0.14 | qua-1 promoter | qua-1 promoter FW +OH to pYPH0.14 | gcgtgtcaataatatcactcatacttttgcactacacggag |
| | | | qua-1 promoter RV +OH to pYPH0.14 | CCATGGCTAAGTCTAGACATcttaaatataggttaagcatgataggat |
| pMM001 | pCFJ150 | luciferase::GFP | unc-54 3'UTR + overhang gfp | GCATGGATGAACTATACAAAgataagagctccgcatcg |
| | | | gfp + overhang unc-54 3'UTR | gccgatgcggagctcttatcTTTGTATAGTTCATCCATGCC |
| | | | luc, piece2 + overhang piece 1 | GACTACAAGgtaagtttaaacagttcggtactaactaacca |
| | | | luc, piece1 + overhang piece 2 | ccgaactgtttaaacttacCTTGTAGTCTTGGAG |
| | | | luc, piece1 + overhang NheI and backbone | tgtcaataatatcactcGCTAGCATGGAGGACGCCAAGAA |
| | | Unc-54 3'UTR | gfp + overhang luc::spacer (piece2) | TACCGGTAGAAAAAATGAGTAAAGGAGAAGAACTTTTCACTGG |
| | | | luc::spacer (piece2) + overhang gfp | GTGAAAAGTTCTTCTCCTTTACTCATTTTTTCTACCGGTAC |
| pMM002 | pMM001 | eft-3 promoter | Peft-3 RV primer (OH to :luciferase) | ATGTTCTTGGCGTCCTCCATtgagcaaagtgtttcccaac |
| | | | Peft-3 FW primer (OH to pCF150) | gcgtgtcaataatatcactcGCACCTTTGGTCTTTTATTGT |

**Supplementary text**

The Hilbert transform can be used to study instantaneous, i.e. time-varying, changes in the amplitude and period of gene expression oscillations. Thus, in contrast to obtaining one value for the period during each of the cycles of oscillations (C1-C4), this approach can reveal how the period changes over time. The temporal resolution is limited by the sample frequency, which is 1 hour for the time courses represented here. Using the Hilbert transform, we observed a relatively constant period in gene expression oscillations during C1-C3 and a gradually increasing period during C4 (Fig 4).

The Hilbert transform, $s_H(t)$, is a linear transformation of signal $s(t)$ to obtain an analytical signal $s_A(t)$,

$$s_A(t) = s(t) + i s_H(t),$$

where $i = \sqrt{-1}$.

The analytical signal is a complex function of time and can be rewritten using Euler's formula, i.e.

$$s_A(t) = A(t)\, e^{i\vartheta(t)},$$

where $A(t)$ and $\vartheta(t)$ are the instantaneous amplitude and the instantaneous phase at time $t$ respectively. The instantaneous frequency is defined as

$$f(t) = \frac{1}{2\pi}\frac{d}{dt}\vartheta(t),$$

and the instantaneous period as

$$T(t) = \frac{1}{f(t)}.$$

(Pikovsky *et al*, 2001)

**References:**


Hendriks G-J, Gaidatzis D, Aeschimann F & Großhans H (2014) Extensive oscillatory gene expression during C. elegans larval development. *Mol. Cell* **53:** 380–392

Lagido C, Pettitt J, Flett A & Glover LA (2008) Bridging the phenotypic gap: Real-time assessment of mitochondrial function and metabolism of the nematode Caenorhabditis elegans. *BMC Physiology* **8:** 7

Olmedo M, Geibel M, Artal-Sanz M & Merrow M (2015) A High-Throughput Method for the Analysis of Larval Developmental Phenotypes in Caenorhabditis elegans. *Genetics* **201:** 443–448

Pikovsky A, Rosenblum M & Kurths J (2001) Synchronization: A universal concept in nonlinear sciences Cambridge: Cambridge University Press

Wu G, Anafi R, Hogenesch J, Hughes M, Kornacker K, Li X & Carlucci M (2019) MetaCycle: Evaluate Periodicity in Large Scale Data. R package version 1.2.0. Available at: https://CRAN.R-project.org/package=MetaCycle

Wu G, Anafi RC, Hughes ME, Kornacker K & Hogenesch JB (2016) MetaCycle: an integrated R package to evaluate periodicity in large scale data. *Bioinformatics* **32:** 3351–3353
