# Supplementary material for: Developmental function and state transitions of a gene expression oscillator in Caenorhabditis elegans
Source: Mol Syst Biol. 2020 Jul 20;16(7):e9498. doi: 10.15252/msb.20209498 (PMC7370751; doi:10.15252/msb.20209498)
Supplement: Supplementary file 2 — Expanded View Figures PDF [file MSB-16-e9498-s002.pdf]

Expanded View Figures

A

	Description	TP	Figure	# osc genes	# osc w/o deviating L4 genes
TC1	early larval time course, replicate 1	1-15	2	3680	NA
TC2	long developmental time course	5-48	4, S1, S4	3739	3448
TC3	TC1 (TP1-13) + TC2 (TP14-48)	1-48	1, 2, 5, 6, 7, S1, S5, S8, S9	3680	3393
TC4	early larval time course, replicate 2	1-24	2	3739	NA
TC5	TC4 (TP1-13) + TC2 (TP14-48)	1-48	S2	3739	NA
TC6	L3-YA TC from Hendriks et al, 2014	21-36	S1	2718	NA
embryo	Hashimshony et al, 2015	10-830	6, S7	3723	3434
dauer	Hendriks et al, 2014	0-15	7	3680	3393

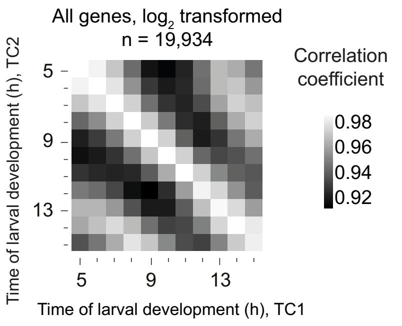

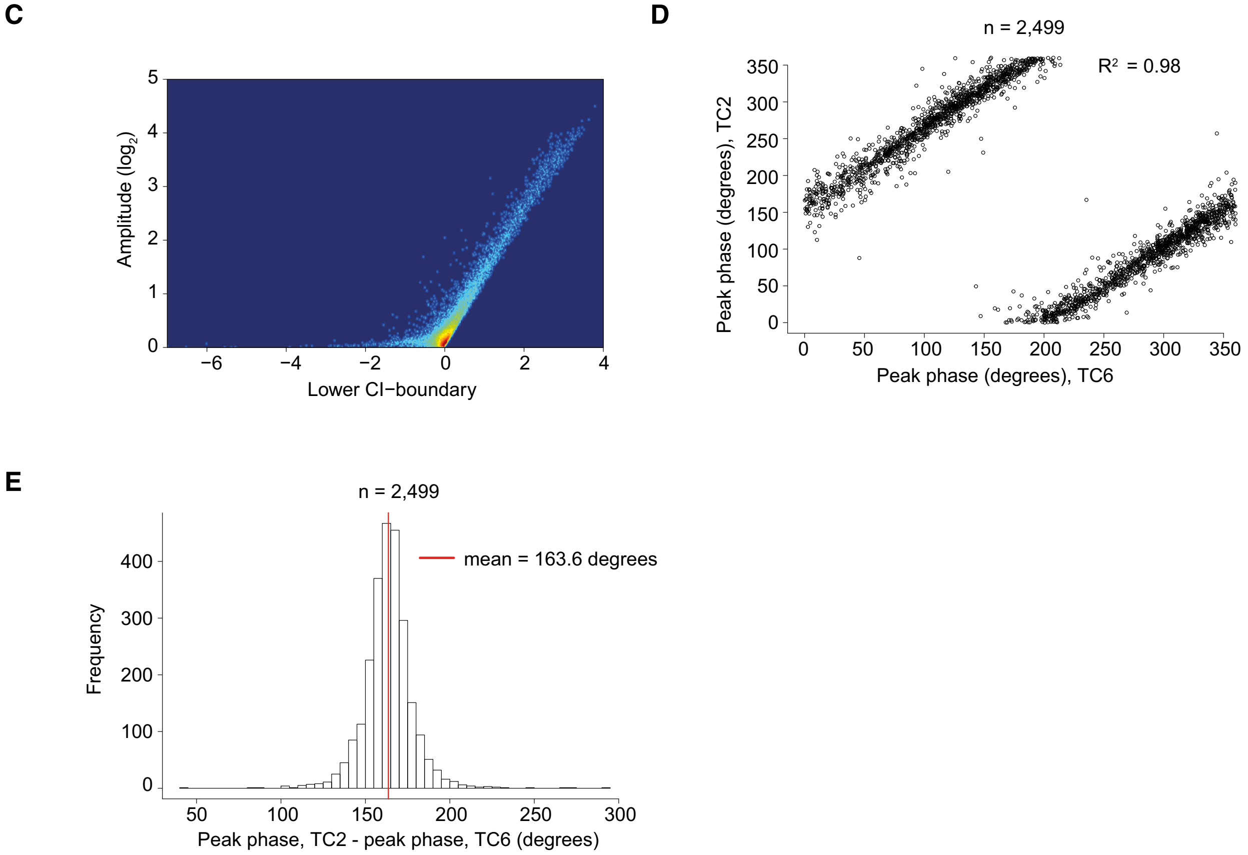

Figure EV1. Identification of 3,739 "oscillating" genes.

A Overview of time courses in this study.

B Pairwise correlation of $\log_2$-transformed count data (n = 19,934) of the early time course (TC1) with the long developmental time course (TC2). High correlation is detected for samples that correspond to the same time points, justifying a fusion of these time courses to one continuous full developmental time course (TC3).

C Smooth scatter of amplitude over lower boundary of 99% confidence interval of the amplitude as determined by cosine fitting and error propagation (see Materials and Methods, related to Fig 1B).

D, E Scatterplot (D) of the peak phase of the long developmental time course (TC2) described here over the previously published L3-YA time course (TC6) (Hendriks *et al*, 2014). Genes that were identified as "oscillating" in both time courses (n = 2,499) are shown. Peak phases correlate well as confirmed by the coefficient of determination, R^2, as indicated. However, they differ systematically (E) because a peak phase of 0° is arbitrarily chosen. A red vertical line indicates the mean phase difference (TC2 – TC6; corrected for circularity as described in Materials and Methods). Note that the gene-specific peak phase calculated here and previously both also differ from the arbitrarily assigned cycle phases in Appendix Fig S7 and their discussion.

© 2020 The Authors

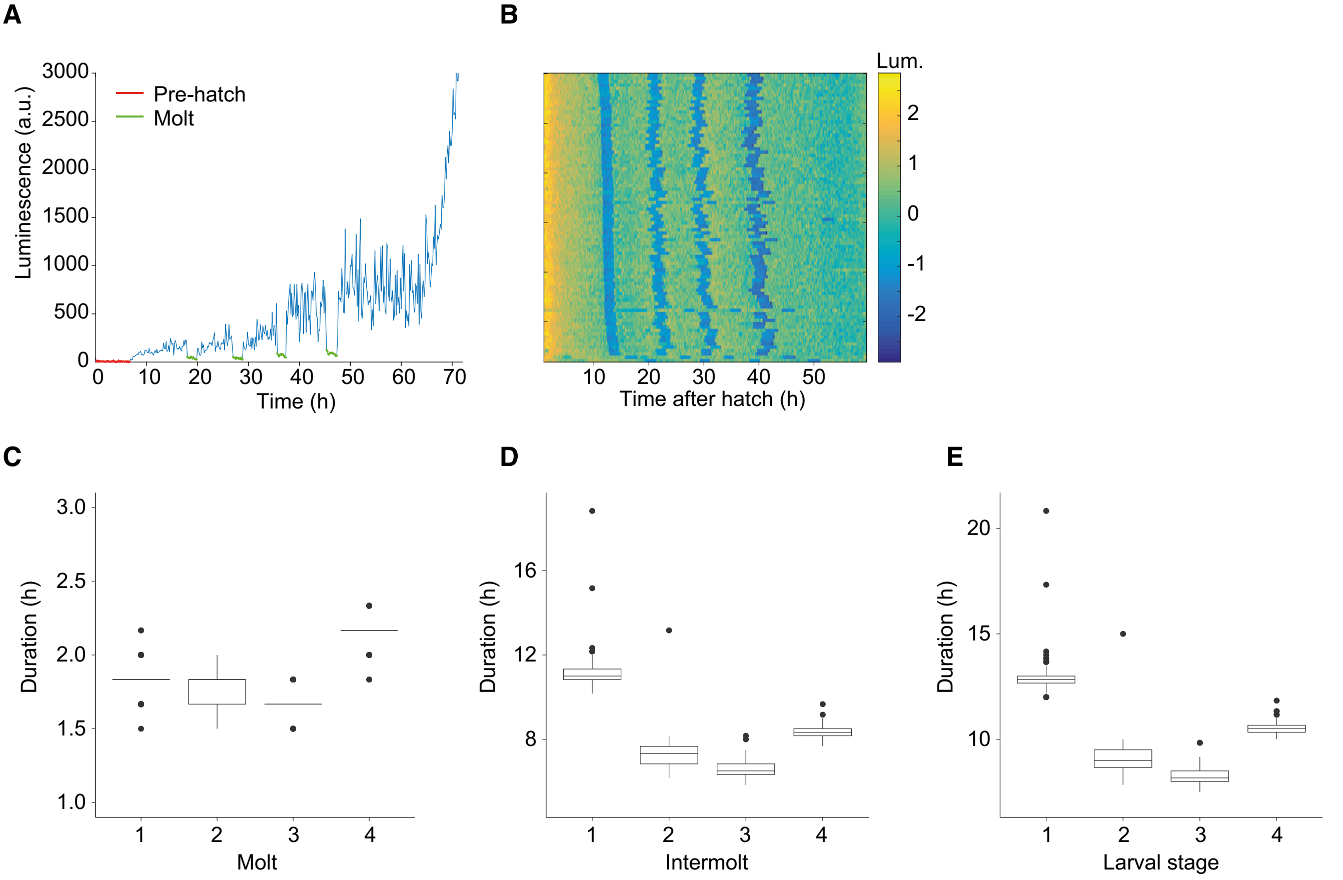

Figure EV2. Quantification of stage durations reveals extended intermolt 1, intermolt 4, and molt 4.

A Representative raw luminescence traces of individual animal (HW1939) grown at 20°C. As the egg-shell is impenetrable to luciferin, a sudden increase in luminescence at the beginning of the time course indicates hatch (pre-hatch in red). Abrupt drops and subsequent rises in luminescence specify molts (in green).

B Heatmap showing trend-corrected luminescence (Lum.) trace for one animal per horizontal line (n = 86). Hatch is set to t = 0 h, and traces are sorted by time of entry into first molt. Blue indicates low luminescence and corresponds to the molts.

C–E Quantification of the duration of each molt (C), intermolt (D), and larval stage (E) in hours for HW1939 animals (n = 86).

Data information: Boxplots in (C–E) extend from first to third quartile with a line at the median, outliers are indicated with a dot, and whiskers show 1.5*IQR.

© 2020 The Authors

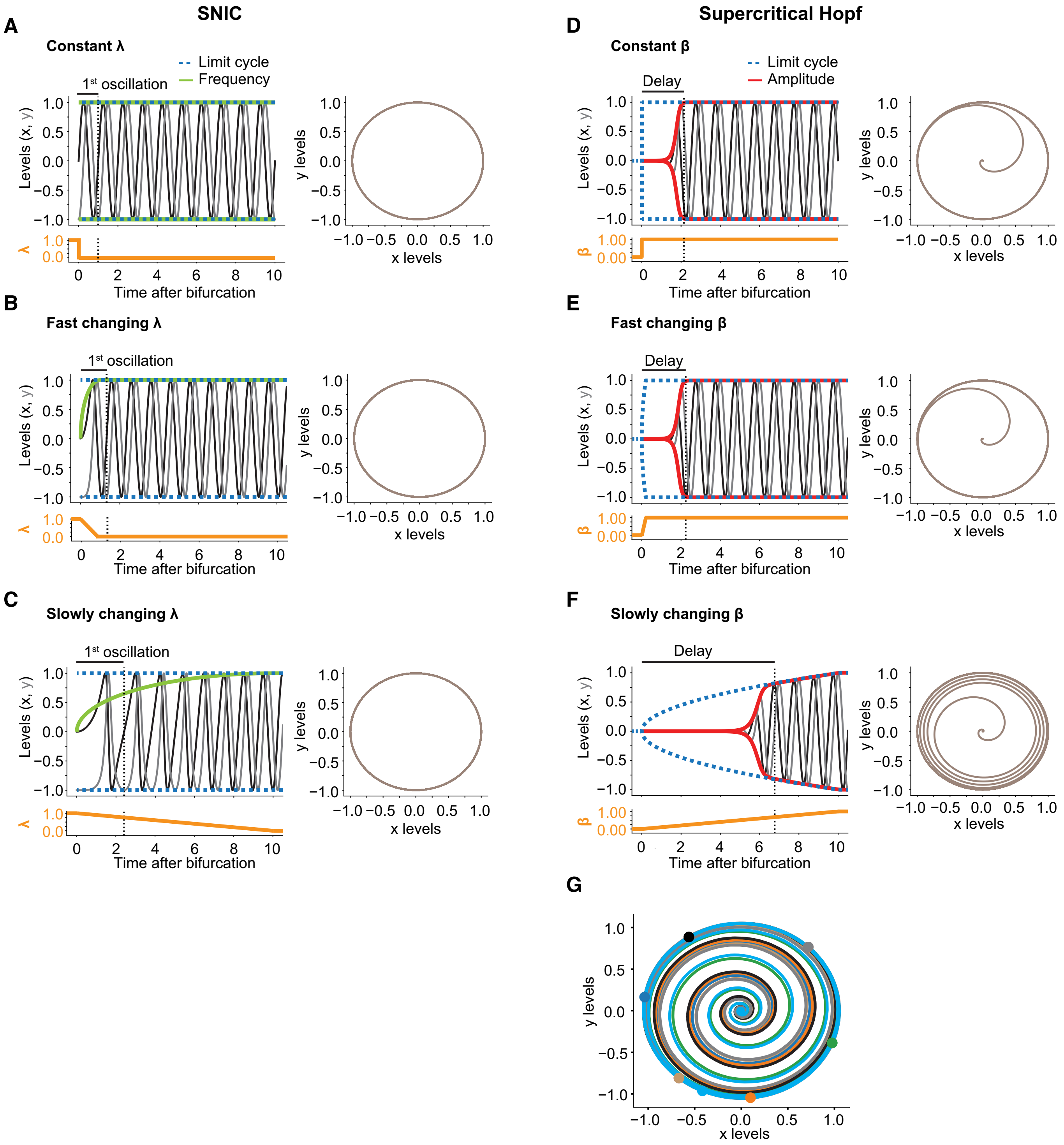

Figure EV3.

 © 2020 The Authors

Figure EV3. Simulations of different bifurcation dynamics for the supercritical Hopf and SNIC bifurcations, respectively.

Simulations were performed for different dynamics of the parameter value using the model described in Materials and Methods. The resulting limit cycle is indicated in blue, the frequency for the SNIC bifurcations in green and the amplitude for the supercritical Hopf bifurcation in red. The change in the respective bifurcation parameter is indicated in the separate plot below in orange.

A–C SNIC bifurcations with their oscillatory behaviors upon instantaneous change to a constant value of λ (A), fast dynamic increase of λ (B), or slowly changing λ (C). The time to complete one oscillation is indicated on the top and in the dotted line, and the oscillatory behavior in the phase space is represented on the right.

D–F Supercritical Hopf bifurcations and resulting oscillatory behaviors upon instantaneous change to a constant value of β (D), fast dynamic increase of β (E), or slowly changing β (F). The delay indicates the time until the amplitude approaches the limit cycle, and the resulting oscillatory behavior is represented in the phase space on the right.

G Stochastic simulations of a supercritical Hopf bifurcation after an instantaneous change to a constant value of β displayed in the phase space. Each iteration had the same initial conditions (x_0 = 0, y_0 = 0, constant β = 1) and additive white noise. The colored dots represent phase at which the individual oscillations reached the limit cycle.

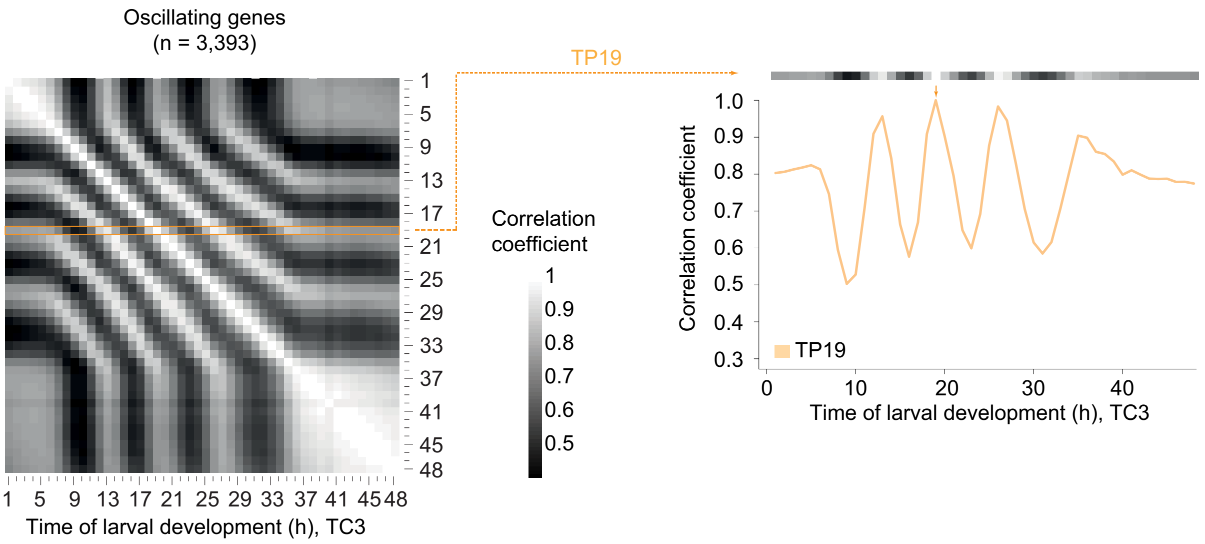

Figure EV4. Correlation line explanation.

Left: Pairwise correlation plot of $\log_2$-transformed oscillatory gene expression patterns without L4 deviating genes obtained from synchronized population of L1 stage larvae at 25°C (TC1, TP1 – 13) combined time points from the long developmental time course (TC2, TP14 –48), as in Fig 5B (*n* = 3,393). Right: The correlation of TP19 versus all other time points is plotted as a line (orange), and correlation with itself at 19 h is 1.0 (orange arrow).

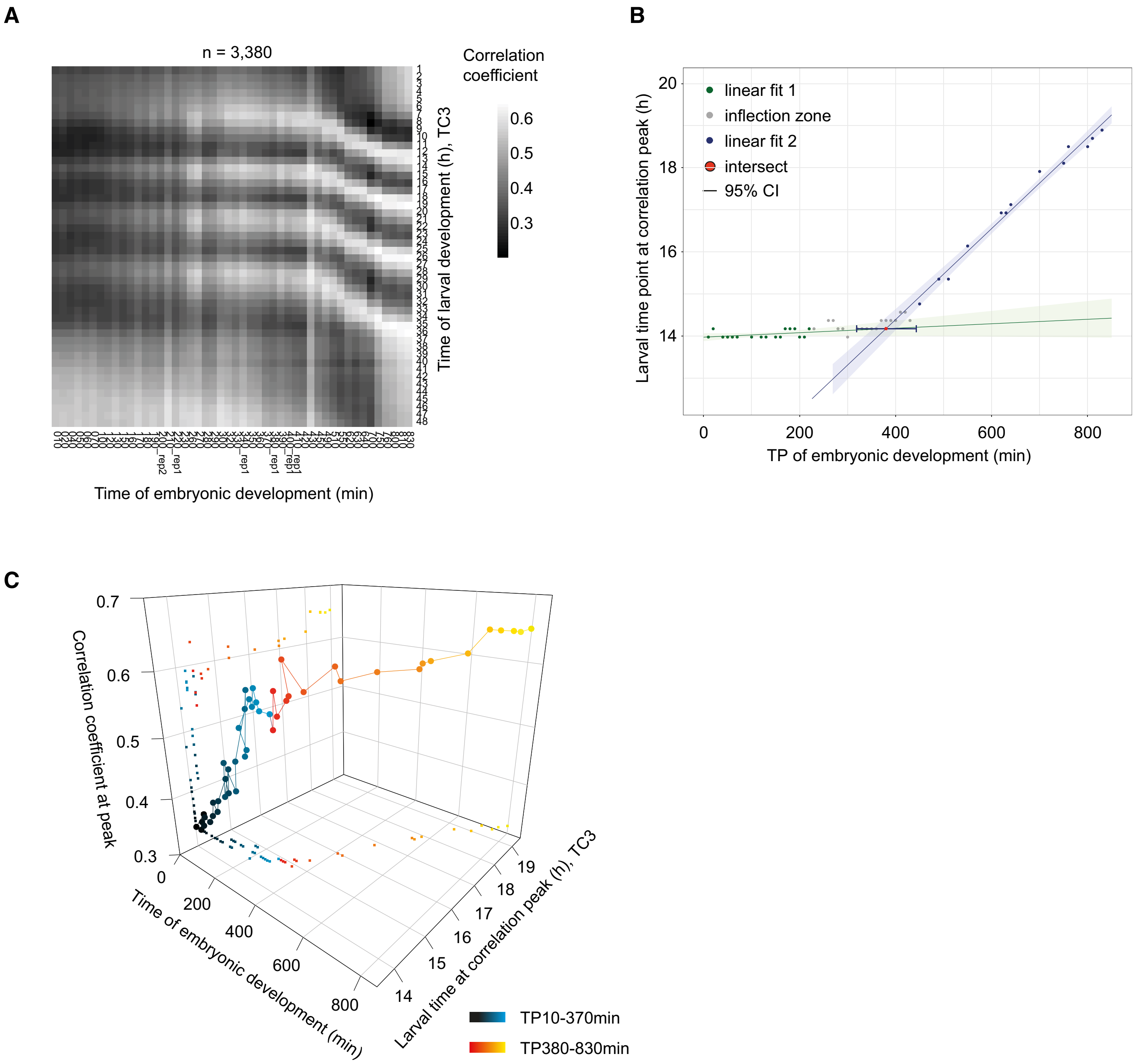

Figure EV5. Initiation of oscillations during mid-embryogenesis.

A Pairwise correlation map of $\log_2$-transformed oscillating gene counts of fused larval time course to embryonic time course (Hashimshony *et al*, 2015).

B Scatter plot showing the larval time point of the larval oscillation cycle 2 (Fig 6) for each embryonic time point. The larval time point of the peak was determined after spline interpolation (9). Linear model 1 (y = 5.312e-04*x + 13.98, P = 0.098, R^2 = 0.162, 16 degrees of freedom) was fitted to the data of embryonic TP10-TP230 min (in green), and linear model 2 (y = 0.0108*x + 10.07, P = 2.08e-11, R^2 = 0.985, 11 degrees of freedom) was fitted to the data of embryonic TP450-TP830 min (in blue). The embryonic time at the intersection (in red, 380.0 min (95%-CI 317.6–444.2 min)) of the linear models was determined in the inflection zone, i.e., points (in grey) not used for model 1 or model 2 fit, and the 95% CI was determined by propagating the standard errors of the coefficients of the linear models (Materials and Methods).

C 3D-scatter plot of the correlation coefficient peak for each embryonic time point to the time of larval development at the second oscillation cycle (C2). Embryonic time is determined by time of sample collection, larval time by spline interpolation. Color scheme as in Fig 6B and C.

 © 2020 The Authors
